# Anesthetic Management of Traumatic Laryngotracheal Laceration in a Postpartum Patient: A Case Report

**DOI:** 10.7759/cureus.109371

**Published:** 2026-05-21

**Authors:** Nistha S Malik, Priyanka Mallya, Abhishek Singh

**Affiliations:** 1 Anesthesiology, All India Institute of Medical Sciences, New Delhi, IND

**Keywords:** difficult airway management, fiberoptic bronchoscopy, neck trauma, self-inflicted injury, videolaryngoscope

## Abstract

Traumatic laryngotracheal lacerations are rare but immediately life-threatening injuries that require rapid, individualized airway management. The anesthesiologist faces the dual challenge of securing a compromised airway while avoiding progression of a partial tear to a complete transection.

We report the case of a 35-year-old woman, 28 days postpartum, who presented to the emergency department after a self-inflicted penetrating anterior neck injury. On arrival, she had hoarseness, moderate respiratory distress, and extensive subcutaneous emphysema. Computed tomography of the neck demonstrated a 5-cm full-thickness defect in the anterior laryngeal wall at the level of the thyroid cartilage, with bilateral paratracheal air pockets and no clear evidence of distal airway disruption. Given the risk of rapid airway deterioration, urgent surgical repair was planned. Airway management was performed while preserving spontaneous ventilation using intravenous fentanyl and incremental inhalational sevoflurane. Videolaryngoscopy (VL) enabled visualization of the glottis, and tracheal intubation was successfully achieved on the first attempt using a gum-elastic bougie and a 7.0-mm cuffed endotracheal tube (ETT). Flexible fiberoptic bronchoscopy (FOB) was then used to advance the tube tip distal to the laceration and confirm tracheobronchial continuity up to the carina. Neuromuscular blockade with intravenous rocuronium was administered only after definitive airway control had been secured, followed by transition to controlled ventilation. Definitive surgical repair proceeded uneventfully. The patient was transferred intubated to the intensive care unit, successfully extubated on postoperative day (POD) 2 following airway assessment, and discharged on POD 6 with minimal residual dysphonia.

This case demonstrates that controlled orotracheal intubation under spontaneous ventilation, with bronchoscopic confirmation, can be safely performed in selected cases of partial laryngeal disruption, in contrast to wound intubation, which is typically reserved for complete transections.

## Introduction

Cut throat injuries, a distinct subset of penetrating neck injuries (PNIs), account for approximately 5%-10% of all traumatic presentations and are associated with multistructural involvement in up to 30% of cases [1,2]. These injuries may result from suicidal, homicidal, or accidental mechanisms and carry a reported mortality rate of 3%-10%, which increases substantially in the presence of laryngotracheal disruption or pharyngoesophageal injury [3]. Mortality is most commonly attributable to hypovolemic shock, acute airway compromise, or septic sequelae following major vascular or aerodigestive tract injury [4]. Prompt identification of “hard signs” is critical, as these mandate immediate intervention. These include active hemorrhage, expanding hematoma, wound bubbling, subcutaneous emphysema, stridor, hoarseness, dysphagia, and associated neurological deficits [5].

The neck is classically divided into three anatomical zones for operative and diagnostic stratification: Zone I (thoracic inlet to cricoid cartilage), Zone II (cricoid cartilage to angle of the mandible), and Zone III (angle of the mandible to the skull base) [6]. Zone II injuries are particularly challenging because of the high density of vital laryngotracheal, vascular, and esophageal structures within a confined space [1]. In the present case, the injury was localized to Zone II, with imaging confirming a full-thickness disruption of the anterior laryngeal wall, constituting a high-risk airway scenario.

Airway management in laryngotracheal trauma remains one of the most complex and high-stakes challenges in anesthesia. Management is guided by several key principles: avoidance of positive-pressure bag-mask ventilation to limit air dissection into soft tissues and worsening airway distortion; avoidance of blind instrumentation to prevent false passage creation or conversion of a partial disruption into a complete transection; and early preparedness for a surgical airway [3]. In cases with extensive open wounds and direct visualization of the distal airway, intubation through the wound has been described as a lifesaving technique [7-10]. However, high-quality evidence guiding airway management strategies in patients with partial laryngotracheal disruption and preserved spontaneous ventilation remains limited. In this setting, a controlled approach using videolaryngoscopy (VL) under maintained spontaneous ventilation, with bronchoscopic confirmation, provides a physiologically sound and reproducible strategy that minimizes the risk of airway collapse and iatrogenic extension of injury.

We report the anesthetic management of a self-inflicted penetrating laryngotracheal injury in a postpartum woman, emphasizing a structured airway strategy, real-time decision-making, and the critical importance of teamwork in achieving favorable outcomes in this rare but life-threatening condition.

## Case presentation

A 35-year-old woman (height 155 cm, weight 66 kg; BMI 27.5 kg/m²), 28 days postpartum, presented to the emergency department following a self-inflicted PNI to the anterior neck with a kitchen knife. A recent psychosocial stressor was identified, as her neonate had been diagnosed with a congenital cardiac anomaly. On arrival, she was conscious, anxious, and able to communicate in short, fragmented sentences while maintaining spontaneous ventilation. A transverse laceration was noted over the anterior neck at the level of the thyroid cartilage (Figure 1).

**Figure 1:**
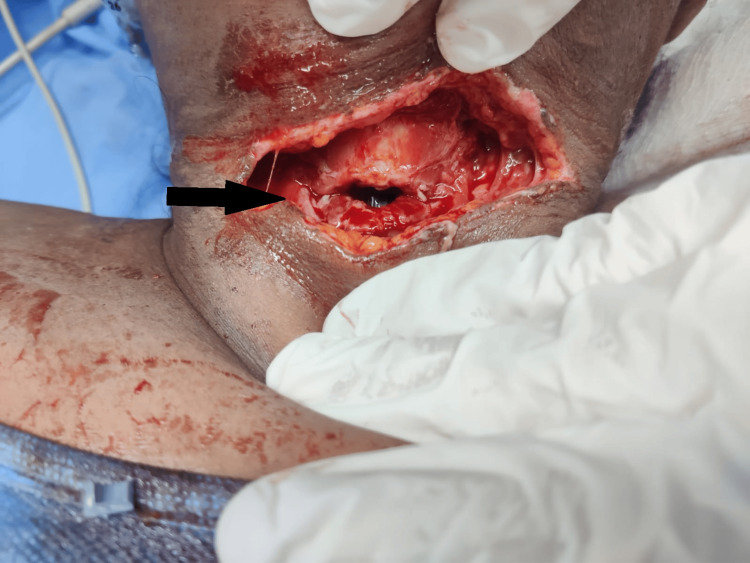
Clinical photograph demonstrating the anterior neck laceration (black arrow), with surrounding soft tissue injury at presentation.

Initial vital signs were as follows: heart rate 100 beats/min, respiratory rate 24 breaths/min, blood pressure 100/60 mmHg, and peripheral oxygen saturation of 97% on room air. Examination revealed hoarseness, moderate respiratory distress, and visible air bubbling from the wound during expiration, consistent with an open airway injury. Palpable crepitus extending across the anterior neck and upper chest indicated extensive subcutaneous emphysema. Focused airway assessment demonstrated adequate mouth opening (>3 finger breadths), Mallampati Class II, a thyromental distance of 6.5 cm, and restricted neck extension secondary to pain. No craniofacial abnormalities or prior history of difficult airway were identified. However, given the disrupted laryngeal framework and active air leak, the airway was preemptively classified as difficult. The presence of air egress through the wound indicated continuity of the airway, thereby excluding complete transection and allowing consideration of controlled orotracheal intubation rather than immediate wound intubation.

In view of her hemodynamic stability, computed tomography (CT) angiography of the neck was performed without delaying definitive airway planning. Imaging revealed a 5-cm defect in the anterior laryngeal wall at the level of the thyroid cartilage, with associated soft tissue disruption. Air tracking was noted along the bilateral paratracheal regions, carotid spaces, and tracheoesophageal groove (Figure 2).

**Figure 2:**
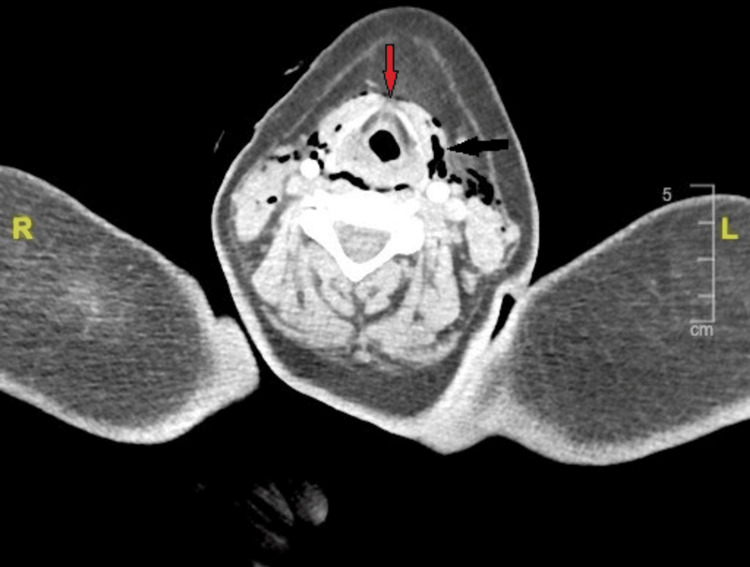
Axial contrast-enhanced CT scan of the neck demonstrating extensive subcutaneous and deep cervical emphysema (black arrow) with anterior laryngeal wall disruption at the level of the thyroid cartilage (red arrow).

No vascular or esophageal injury was identified. A multidisciplinary preprocedural briefing involving the anesthesiology and trauma surgery teams was conducted. The primary airway strategy was induction under spontaneous ventilation followed by VL-guided intubation using a bougie. Awake fiberoptic bronchoscopy (FOB) was designated as the backup approach, while an emergency surgical airway (tracheostomy or cricothyrotomy) was prepared as the rescue plan. A difficult airway cart was kept immediately available, and the surgical team remained present in the operating room throughout airway management. Positive-pressure bag-mask ventilation and cricoid pressure were deliberately avoided.

Standard monitoring, including electrocardiography, noninvasive blood pressure monitoring, pulse oximetry, capnography, and temperature monitoring, was instituted. Adequate intravenous access was secured, and blood samples were sent for crossmatching. Preoxygenation was performed with 100% oxygen for three minutes, targeting an end-tidal oxygen fraction of >90% to optimize safe apnea duration. Anesthesia was induced while preserving spontaneous ventilation. Intravenous fentanyl 100 µg was administered, and sevoflurane (2%-5%) in 100% oxygen was titrated to achieve adequate anesthetic depth without compromising respiratory drive. Ketamine was prepared as an alternative induction agent because of its favorable profile in maintaining airway tone and spontaneous respiration. VL using a C-MAC VL (Karl Storz SE & Co. KG, Tuttlingen, Germany) with a D-blade provided a partial glottic view (percentage of glottic opening ≈50%), limited by blood and mucosal edema. A gum-elastic bougie was gently advanced through the glottic aperture, and a 7.0-mm cuffed endotracheal tube (ETT) was railroaded successfully on the first attempt (Figure 3).

**Figure 3:**
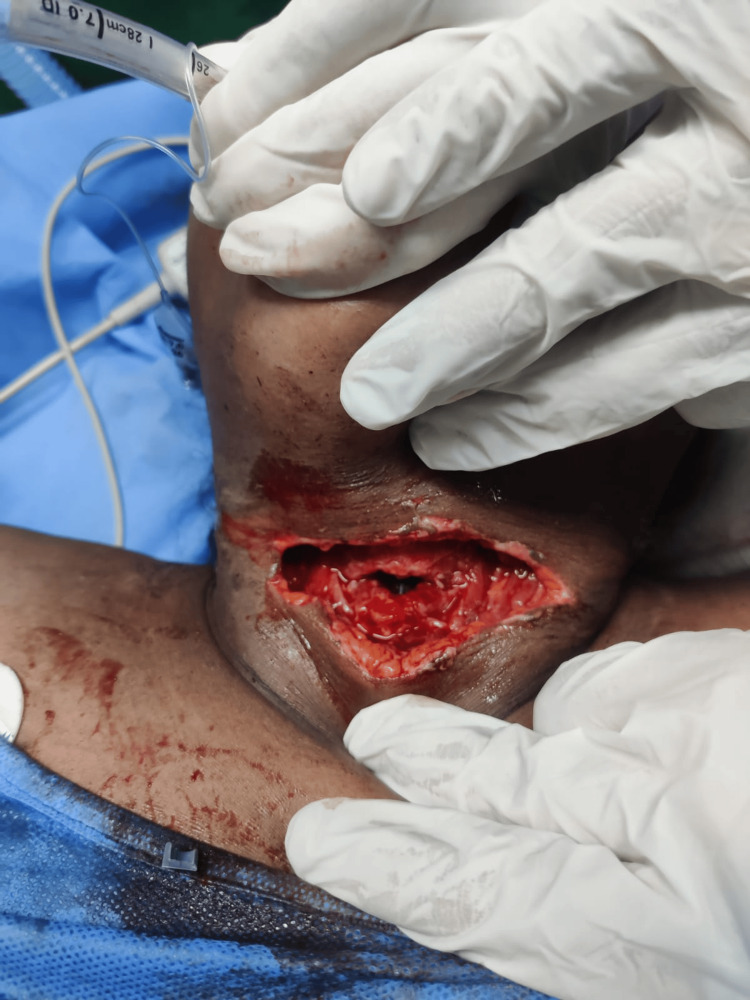
Intraoperative clinical image demonstrating orotracheal intubation in a patient with a self-inflicted penetrating laryngotracheal injury.

Flexible FOB was then introduced through the ETT, allowing controlled advancement of the tube distal to the laceration under direct visualization, thereby minimizing the risk of false passage or extension of the injury. It also confirmed continuity of the tracheobronchial tree up to the carina, excluding associated distal airway injury (Figure 4).

**Figure 4:**
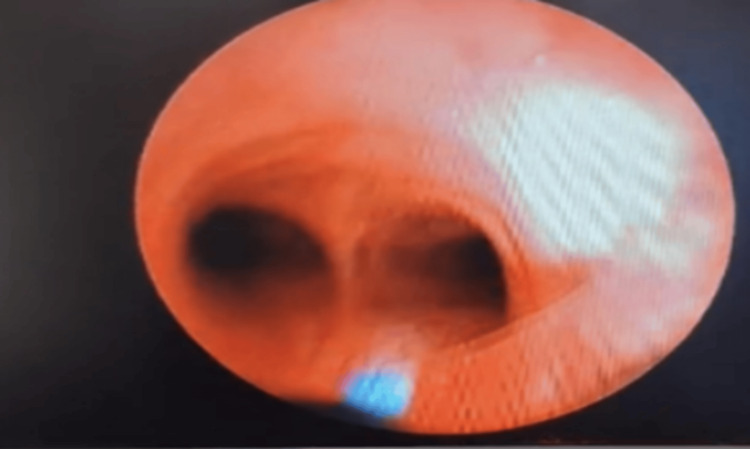
Intraoperative flexible fiberoptic bronchoscopy image confirming an intact airway, with no mucosal injury visualized to the level of the carina following endotracheal tube placement distal to the laryngeal defect.

Neuromuscular blockade with intravenous rocuronium was administered only after confirmation of secure airway positioning and adequate ventilation. Definitive surgical repair consisted of primary closure of the thyroid cartilage defect with layered soft tissue reconstruction and placement of a suction drain. Intraoperative evaluation confirmed intact esophageal and neurovascular structures. The intraoperative course remained stable, without hypoxemia, airway complications, or hemodynamic instability. The patient was transferred to the intensive care unit (ICU) intubated. She was successfully extubated on POD 2 following clinical and airway assessment, with minimal residual hoarseness. Enteral nutrition was advanced from nasogastric feeding to supervised oral intake on POD 4 without dysphagia. Given the self-inflicted nature of the injury in the postpartum period, psychiatric evaluation and counseling were initiated. The patient was discharged in stable condition on POD 6.

## Discussion

This case illustrates the anesthetic management of a confirmed open Zone II laryngotracheal laceration, one of the most challenging airway emergencies in clinical anesthesia. The central dilemma is securing a disrupted airway while avoiding progression of a partial injury to a complete transection.

Classification and initial assessment

Zone II is the most frequently involved region in PNIs, accounting for up to 86.6% of cases in published series, and is particularly challenging due to the high density of laryngotracheal, vascular, and esophageal structures [1,11,12]. The presence of “hard signs,” such as air bubbling from the wound, subcutaneous emphysema, hoarseness, and dyspnea, clearly classified our patient as requiring emergent airway control and operative intervention. In this case, hemodynamic stability allowed for CT angiography, which delineated the location, extent, and depth of the laryngeal defect while excluding associated vascular injury. This approach aligns with contemporary evidence supporting selective, imaging-based evaluation in stable patients with PNIs [13]. However, imaging should not delay definitive management or preparation for emergent airway control [14].

Airway strategy

Airway management in confirmed laryngotracheal disruption differs fundamentally from conventional difficult airway scenarios and must adhere to specific principles. The key tenets described by Youssef and Raymer [3] were applied in our case. First, bag-mask ventilation was deliberately avoided because positive-pressure ventilation in the presence of an open airway injury may force air into the paratracheal soft-tissue planes, resulting in pneumomediastinum, worsening subcutaneous emphysema, and further distortion of airway anatomy [3]. Second, blind advancement of an ETT was avoided, as this risks entry into the laryngeal defect, creation of a false passage, or conversion of a partial injury into a complete transection [3]. Third, a surgical airway was prepared in advance, with the team fully briefed prior to induction [3].

In contrast, when the laryngotracheal defect is extensive and the distal trachea is directly visualized, intubation through the wound represents the most expedient lifesaving approach. This strategy has been described in cases of complete airway disruption, where the ETT was inserted directly through the open airway under visual guidance [3,7,8]. In such scenarios, careful advancement under direct visualization is essential to avoid further extension of the injury. In the present case, the presence of partial laryngeal disruption with preserved spontaneous ventilation favored a controlled orotracheal intubation as the first-line approach, with immediate readiness to convert to wound intubation or a surgical airway if required.

Spontaneous vs controlled ventilation

The decision to maintain spontaneous ventilation during induction is widely supported in PNIs [3,12]. Neuromuscular blockade abolishes spontaneous respiratory effort and, if intubation fails, leaves the clinician with no airway and an apneic patient. Furthermore, muscle paralysis eliminates the protective reflexes that may help maintain residual airway patency in the setting of partial laryngeal disruption [3]. Sevoflurane was selected for its rapid onset, ease of titration to achieve a dose-dependent depth of anesthesia, and its ability to preserve spontaneous ventilation during induction.

This approach contrasts with that of Meena et al. [11], who used a modified rapid sequence induction (RSI) with VL in a patient with a knife in situ and no CT or clinical evidence of airway breach. In that case, the intact airway justified RSI; in our patient, the confirmed 5-cm full-thickness laryngeal defect made RSI inappropriate [3,11]. Similarly, Góis et al. [7] used ketamine-propofol-fentanyl sedation rather than full RSI, preserving spontaneous ventilation, given the severity of airway disruption. The choice of induction technique must therefore be individualized to the specific injury pattern and not applied as a blanket protocol to all PNIs. In our case, neuromuscular blocking agents were withheld until ETT placement distal to the injury was confirmed by FOB, and ventilation was deemed adequate. This is a cardinal principle endorsed across multiple published series [3,7,8].

VL, bougie, and FOB: a combined approach

A combined approach using VL, a gum-elastic bougie, and flexible FOB offers complementary advantages that directly address the anatomical and physiological challenges of laryngotracheal disruption. VL provides a magnified view of the glottis without requiring alignment of the oral, pharyngeal, and laryngeal axes. This is particularly advantageous in the presence of airway distortion, edema, or active bleeding, where conventional direct laryngoscopy may be limited. The use of a hyperangulated blade further enhances glottic visualization in anatomically disrupted airways while minimizing the need for excessive force, thereby reducing the risk of exacerbating the injury. The bougie facilitates atraumatic navigation through a partially visualized or narrowed glottic opening, improving first-pass success while minimizing repeated instrumentation. FOB adds a critical safety layer by enabling controlled advancement of the ETT beyond the site of injury under direct vision, thereby preventing false passage or extension of the laceration, while also confirming tracheobronchial continuity up to the carina. Importantly, in our patient, FOB was not used as the primary intubation modality, as blood, secretions, and mucosal edema can significantly impair visualization in emergency settings; in such circumstances, VL or direct wound access should be prioritized over persistence with a failing fiberoptic technique [3]. Together, this stepwise strategy (VL for visualization, bougie for guided placement, and FOB for confirmation) minimizes airway trauma, preserves physiological stability, and offers a reproducible, controlled approach for managing partial laryngotracheal disruption.

Ventilatory strategy and extubation

In selected patients, particularly those with significant airway edema, extensive surgical manipulation, or physiological instability, elective continuation of postoperative mechanical ventilation is appropriate [11]. In our case, delayed extubation was performed on POD 2 using intravenous lignocaine to attenuate airway reflexes. The rationale for controlled extubation is to avoid vigorous coughing or straining, which can generate marked intrathoracic pressure fluctuations and risk disruption of the laryngeal repair; therefore, a smooth and hemodynamically stable emergence is essential [11]. Anticipation of difficult reintubation is equally critical, as postoperative airway edema, hematoma formation, and altered anatomy may render reintubation significantly more challenging than the initial airway management.

Multidisciplinary coordination

The management of open PNIs requires real-time, structured collaboration among the anesthesiologist, trauma surgeon, intensivist, and, in cases of self-inflicted injury, the psychiatrist [1,4,11]. Beyond technical expertise, outcomes depend critically on non-technical skills, including effective communication, shared mental models, clearly defined roles, and predefined escalation pathways [7]. In our case, a preprocedural team briefing with explicitly agreed rescue strategies, along with the continuous presence of the surgical team from the onset of induction, was central to the safe and coordinated conduct of airway management.

## Conclusions

We report the successful anesthetic management of a confirmed open Zone II laryngotracheal laceration in a postpartum woman using a structured, spontaneous ventilation-based induction strategy with VL, bougie-assisted intubation, and confirmatory FOB. Several core principles guided our airway management, including avoidance of positive-pressure bag-mask ventilation and blind instrumentation, maintenance of spontaneous ventilation until definitive airway control was achieved, deferral of neuromuscular blockade until bronchoscopic confirmation of tube position, and continuous readiness for immediate surgical airway access. This case emphasizes that airway strategy in PNIs must be individualized according to the pattern and severity of injury. The choice between direct wound intubation and controlled orotracheal intubation depends on the degree of airway disruption, accessibility of the distal trachea, and the patient’s physiological status. Early diagnosis, meticulous planning, and coordinated multidisciplinary management remain the key determinants of favorable outcomes.
